# Biogenesis and function of the mitochondrial solute carrier (SLC25) family in yeast

**DOI:** 10.1515/hsz-2025-0152

**Published:** 2025-06-24

**Authors:** Celina Nauerz, Ophry Pines, Johannes M. Herrmann

**Affiliations:** Cell Biology, 26562 University of Kaiserslautern, RPTU, Erwin-Schrödinger-Strasse 13, D-67663 Kaiserslautern, Germany; Microbiology and Genetics, IMRIC, Faculty of Medicine, Hebrew University of Jerusalem, Jerusalem, Israel

**Keywords:** membrane transport, metabolism, mitochondria, protein translocation, TIM22 complex

## Abstract

The mitochondrial solute carrier family, also called SLC25 family, comprises a group of structurally and evolutionary related transporters that are embedded in the mitochondrial inner membrane. About 35 and 53 mitochondrial carrier proteins are known in yeast and human cells, respectively, which transport nucleotides, metabolites, amino acids, fatty acids, inorganic ions and cofactors across the inner membrane. They are proposed to function by a common rocker-switch mechanism, alternating between conformations that expose substrate-binding pockets to the intermembrane space (cytoplasmic state) and to the matrix (matrix state). The substrate specificities of both states differ so that carriers can operate as antiporters, symporters or uniporters. Carrier proteins share a characteristic structure comprising six transmembrane domains and expose both termini to the intermembrane space. Most carriers lack N-terminal presequences but use carrier-specific internal targeting signals that direct them into mitochondria via a specific import route, known as the ‘carrier pathway’. Owing to their hydrophobicity and aggregation-prone nature, the mistargeting of carriers can lead to severe proteotoxic stress and diseases. In this review article, we provide an overview about the structure, biogenesis and physiology of carrier proteins, focusing on baker’s yeast where their biology is particularly well characterized.

## 1 Introduction: the mitochondrial solute carrier (SLC25) family

**Figure 1:**
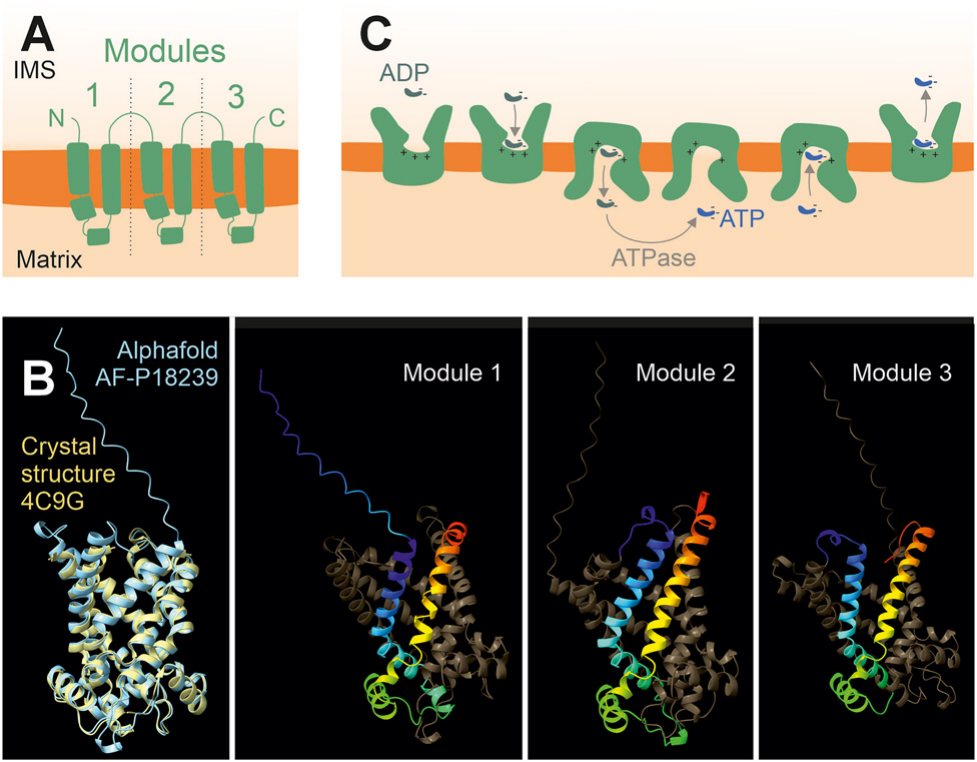
Structure and biochemical properties of mitochondrial SLC25 carrier proteins. (A) Structural overview revealing the three repetitive modules of carriers, each forming a hairpin-like structure. (B) The crystal structure of the ATP/ADP carrier Pet9 (shown in yellow) has been solved experimentally (Ruprecht et al. 2014) and is almost identical to the structure predicted by Alphafold 2 (shown in blue) (Jumper et al. 2021). The Alphafold structure also shows the linker and terminal regions that were not resolved in the crystal structure. However, the information from the crystal structures are very informative as they were solved from complexes with inhibitors which had locked Pet9 in its two functional states, exposing the substrate-binding sites to the cytosol (IMS) and the matrix. The three snapshots show the three modules of the Pet9 Alphafold structure in rainbow coloring generated by Chimera X 1.9 (Pettersen et al. 2004). (C) Schematic reaction cycle of the ATP/ADP antiport driven by Pet9 (Ruprecht et al. 2019).

Mitochondria are essential organelles which make up about 10–25 % of the volume of eukaryotic cells, depending on the cell type and metabolic conditions. They play crucial roles in energy production, i.e. the regeneration from ATP from ADP and phosphate, but also in many other metabolic pathways including the biogenesis of amino acids, lipids, heme, lipoic acid, iron-sulfur clusters or ubiquinone. Most of these pathways involve reactions that are carried out in cooperation with enzymes of the mitochondrial matrix as well as enzymes of the cytosol. The metabolites can easily diffuse through the beta-barrel proteins of the outer mitochondrial membrane (called porins, and the voltage-gated anion channel, VDAC, in humans). However, the inner membrane is impermeable to these metabolites. Metabolite transport across the inner membrane is mediated by a large family of specific transporters, most of which are members of the mitochondrial solute carrier (or SLC25) family. In this review article we will refer to such mitochondrial solute carrier proteins as ‘carriers’ (Palmieri et al. 2000; Palmieri and Monne 2016; Ruprecht and Kunji 2020). Members of this family were identified in baker’s yeast and human, 35 and 53 respectively, which share a common structural organization (Figure 1A). These carriers function as monomers (Bamber et al. 2007; Kunji and Crichton 2010; Ruprecht et al. 2019) but can be associated with other inner membrane complexes such as protein translocases or respiratory chain enzymes (Cimadamore-Werthein et al. 2024; Dienhart and Stuart 2008), however, the information and physiological significance of such interactions is very limited. The abundance of individual carriers differs considerably: the ATP/ADP carrier Pet9 (alias Aac2) is one of the most abundant inner membrane proteins of yeast mitochondria while some other carriers are at a very low abundance, present in only a few copies per cell (Morgenstern et al. 2017, 2021) (Table 1). The abundance and expression of carriers can depend on prevailing metabolic conditions.

**Table 1:** Mitochondrial carriers of *S. cerevisiae.*

Name	Human homolog	AA, abundance^a^	Physiological role
Aac1, YMR056c	SLC25A4/A5/A6/A31 (also AAC1-4)	309, 28,000	ATP/ADP antiporter
Aac2 (Pet9), YBL030c		318, 190,000	ATP/ADP antiporter
Aac3, YBR085w		307, ?	ATP/ADP antiporter
Agc1, YPR021c	SLC25A12/A13 (AGC1/2)	902, 117	Aspartate/glutamate carrier
Ant1, YPR128c	SLC25A17	328, 180	Peroxisomes, ATP/ADP resp. ATP/AMP antiporter
Crc1, YOR100c	SLC25A20 (CAC)	327, 9,000	Carnitine carrier
Ctp1, YBR291c	SLC25A1 (CIC)	299, 372	Citrate carrier
Dic1, YLR348c	SLC25A10 (DIC)	298, 4,000	Dicarboxylate carrier
Flx1, YIL134w	SLC25A32	311, ?	FAD transporter
Ggc1(Yhm1), YDL198c	?	300, 9,000	GTP/GDP carrier
Hem25, YDL119c	SLC25A38 (GlyC)	307, 117	Glycine carrier, involved in heme biosynthesis
Leu5, YHR002w	SLC25A42	357, ?	Coenzyme A carrier
Mir1, YJR077c	SLC25A3 (PiC)	311, 102,000	Phosphate
Mme1, YMR166c	?	368, ?	Mg2+ exporter
Mrs3, YJL133w	SLC25A37/28 (MFRN1/2)	314, 95	Fe2+ carrier
Mrs4, YKR052c		304, 170	
Mrx20, YFR045w	?	309, ?	Unknown
Mrx21, YPR011c	SLC25A42	326, ?	Adenosine 5′-phosphosulfate carrier
Mtm1, YGR257c	?	366, 512	Pyridoxal 5′ -phosphate
Ndt1, YIL006w	SLC25A51/A52 (MCART1/2)	373, ?	NAD^+^ carriers
Ndt2, YEL006w		335, 35	
Oac1, YKL120w	SLC25A34/A35	324, 2,600	Oxaloacetate-sulfate carrier
Odc1, YPL134c	SLC25A21 (Odc)	310, 18,000	Oxodicarboxylate carriers
Odc2, YOR222w		307, 4,500	
Ort1, YOR130c	SLC25A15/A2 (ORC1/2)	292, 308	Ornithine carrier
Pic2, YER053c	SLC25A3 (PiC)	300, 4,300	Phosphate carrier
Rim2, YBR192w	SLC25A33/A36 (PNC1/2)	377, 330	Pyrimidine nucleotide carrier
Sal1, YNL083w	SLC25A24/A23/A25/A41	494, ?	ATP-Mg/Pi carrier
Sam5 (Pet8), YNL003c	SLC25A26 (SAMC)	284, 1,500	S-adenosylmethionine carriers
Sfc1, YJR095w	?	322, 40,000	Succinate–fumarate carrier
Tpc1, YGR096w	SLC25A19 (TPC)	314, 34	Thiamine pyrophosphate carriers
Ugo1, YDR470c	SLC25A43	502, 411	Outer membrane, required for mitochondrial fusion eand lipid transfer from the ER
Yhm2, YMR241w		314, 19,000	Citrate-oxoglutarate carrier
Ymc1, YPR058w	SLCA45/A48/A47/A29	307, 3,400	Glutamate carrier
Ymc2, YBR104w	SLCA45/A48/A47/A29	329, 108	Glutamate carrier

Note that yeast and human homologs might differ in their substrate spectrum and physiological relevance. ^a^For abundance the mean copy number of glycerol-grown cells is shown based on proteomics data (Morgenstern et al. 2017).

Mitochondrial carriers are largely considered to have evolved within eukaryotes and are generally absent from bacteria. Whereas most members of the carrier family reside in the mitochondrial inner membrane, there are some exceptions: In yeast, the Ant1 protein is functionally similar to the ATP/ADP carrier Pet9, however, it is located in peroxisomes and functional in peroxisomal fatty acid oxidation; it presumably functions as an ATP/AMP exchanger (Palmieri et al. 2001b; van Roermund et al. 2001). The human protein PMP34 (SLC25A17) has an similar role in peroxisomal physiology (Visser et al. 2002). Carrier-derived proteins are also found in the mitochondrial outer membrane, even though these carrier-like proteins do not contain the full 6-transmembrane structure, compatible with polar metabolite transport. The yeast protein Ugo1 (as well as its human ortholog SLC25A46) plays a role in mitochondrial fusion and lipid transfer from the endoplasmic reticulum (ER) (Coonrod et al. 2007; Janer et al. 2016; Sesaki and Jensen 2001; Steffen et al. 2017; Schuettpelz et al. 2023). MTCH1 and MTCH2 are two additional carrier-related proteins in the outer membrane of mammalian mitochondria, where they facilitate the insertion of tail-anchored proteins into the lipid bilayer and facilitate lipid metabolism (Chourasia et al. 2025; Guna et al. 2022; Labbe et al. 2021). All other carriers reside in the mitochondrial inner membrane. In this review, we will describe the structure, biogenesis and function of these inner membrane carriers of yeast mitochondria.

## 2 The structure of carriers

Mitochondrial carriers are between 30 and 35 kDa in mass and consist of three repetitive ‘carrier modules’ each containing two transmembrane domains that are separated by a hydrophilic matrix-exposed loop (Figure 1A). Each carrier module is about 100 residues long and contains a conserved carrier signature motif (Px[D/E]xx[K/R]x[K/R]x_20–30_[D/E]Gx_4_[W/Y/F][K/R]G) (Palmieri 1994). Despite the overall similarity of their sequences, different subclasses can be distinguished based on their sequence, substrate specificity and physiological role (Byrne et al. 2023; Monne et al. 2023).

The structures of several carrier proteins were recently solved, including the yeast carriers Pet9 and Aac3 (Ruprecht et al. 2014, 2019). By locking their conformation with the inhibitors carboxyatractolyside and bongkrekic acid, it was possible to get high-resolution images of the cytoplasmic and the matrix state, respectively. These structures demonstrate that the six transmembrane domains form a cylindrical protein in the membrane with threefold pseudo-symmetry (Figure 1B). Carriers constitute a central opening to the intermembrane space (cytoplasmic state) or the matrix (matrix state) with a central, deeply membrane-embedded substrate-binding site for ATP or ADP, respectively (Nury et al. 2006; Pebay-Peyroula et al. 2003; Ruprecht et al. 2014, 2019). Salt bridges form a cytoplasmic network and a matrix network, stabilizing these two alternative conformations (Figure 1C). These characteristic salt bridges are formed by the carrier signature motifs (Ruprecht and Kunji 2020).

For translocation, the carriers switch between these two conformations constituting a rocker-switch mechanism in which one substrate molecule is translocated at a time. Charges on the surface and in the binding pocket define the substrate spectrum and serve as counter-ions to facilitate the translocation of charged substrates such as ATP or ADP (Figure 1C). Thereby, even the transport of negatively charged ADP against the membrane potential of the inner membrane (towards the negatively charged matrix side), becomes possible. The overall mechanism seems to be conserved among all carriers and even the uncoupling protein UCP1 (SLC25A7) which facilitates thermogenesis in brown adipose tissues of animals, shares these functional elements and operates by a comparable carrier-like mechanism (Jones et al. 2023, 2024; Kang and Chen 2023).

## 3 The biogenesis of carriers

Mitochondria contain their own genome which encodes a small number of very hydrophobic subunits of the respiratory chain and the ATP synthase (Ott et al. 2016). Carrier-encoding genes are not found in mitochondrial genomes, not even in ‘primitive’ protists which still contain a large set of mitochondrial genes. This is consistent with the understanding that carriers are of eukaryotic origin (Gray et al. 2020). Hence, all carrier proteins are nuclear encoded and synthesized in the cytosol. However, in contrast to most other proteins of the matrix and the inner membrane, carrier proteins lack N-terminal presequences that serve as mitochondrial targeting signals. Carriers employ the translocase of the outer membrane (TOM) complex and the TIM22 complex, the inner membrane translocase (not the inner membrane TIM23 complex; Sirrenberg et al. 1996). The different pathways that direct proteins into the outer membrane, the intermembrane space, the inner membrane and the matrix had been described in depth in a series of review articles (Araiso et al. 2022; Herrmann and Bykov 2023; Horten et al. 2020; Wiedemann and Pfanner 2017).

Carrier proteins contain internal targeting information as a part of their mature structure. Thereby, each carrier module contains targeting information so that the targeting signals are partially redundant (Brandner et al. 2005). However, their ability to promote the translocation across the TOM channel and the insertion into the inner membrane may differ. For the carrier proteins that have been analyzed in more detail, it appears that the most C-terminal module is of particular importance for their efficient targeting and biogenesis (Brandner et al. 2005; Sirrenberg et al. 1996; Wiedemann et al. 2001). Worth stating is that due to their very hydrophobic nature, carrier proteins are bound by chaperones in the cytosol which keep them in an import-competent confirmation and prevent their mistargeting to the ER (Opalinski et al. 2018; Young et al. 2003; Xiao et al. 2021). Hsp70 and Hsp90 chaperones as well as several of the co-chaperones presumably help to “usher” carriers to the mitochondrial surface but a detailed description of this awaits further research (Bykov et al. 2020; Zara et al. 2009).

**Figure 2:**
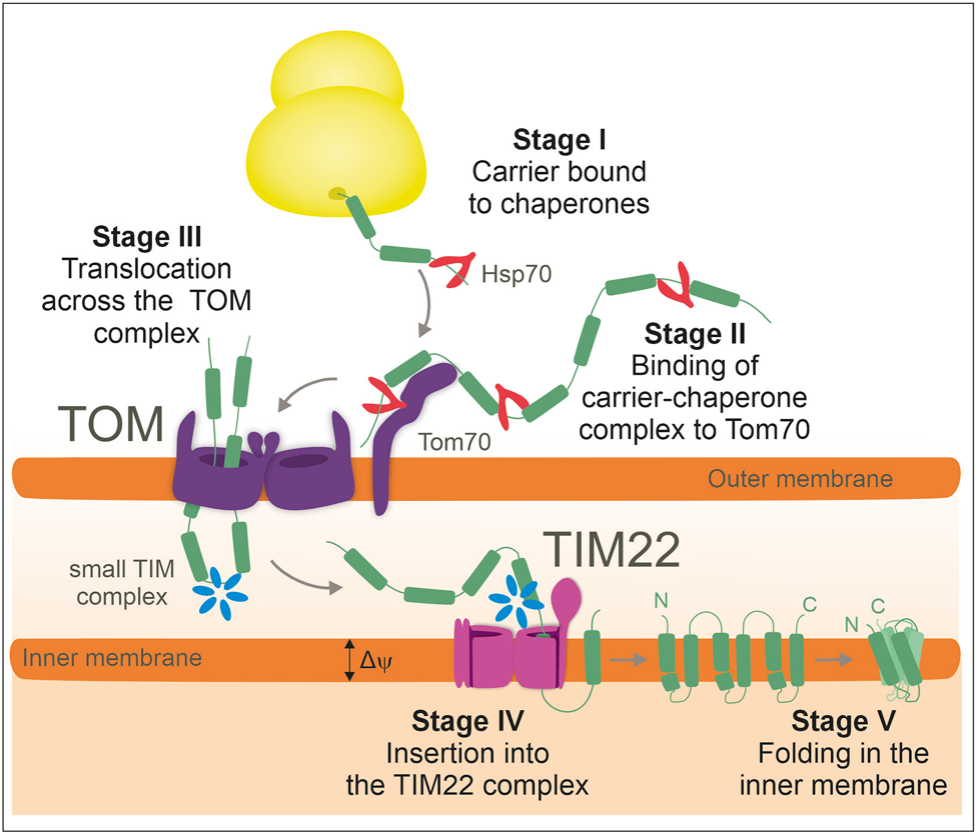
The carrier import pathway. The import pathway of carrier proteins can be divided into five distinct stages as depicted. Cytosolic chaperones keep carrier proteins import-competent and facilitate the binding to the Tom70 receptor. Following translocation through the TOM complex, small Tim complexes serve as chaperones to facilitate transfer of hydrophobic carrier proteins into the TIM22 complex. The TIM22 complex mediates the lateral insertion of carriers into the inner membrane where they fold into their final monomeric structure.

The biogenesis of carriers can be separated into five distinct stages (Figure 2). The internal targeting signals of the carrier modules are recognized by the outer membrane receptor Tom70. Tom70 contains an additional binding site for Hsp70 and Hsp90 which is assumed to increase its affinity for carrier-chaperone complexes (Backes et al. 2021; Young et al. 2003). From Tom70, the carriers are passed on to the protein conducting pore of the TOM complex through which they translocate in a hairpin-like conformation (Söllner et al. 1990; Wiedemann et al. 2001). In the IMS, soluble hexamers of small Tim proteins (Tim9/Tim10 or Tim8/Tim13) bind to these incoming carrier proteins and “escort” them to the TIM22 complex of the inner membrane (Curran et al. 2002a,b; Koehler et al. 1998; Luciano et al. 2001). The ring-like small Tim complexes expose a highly conserved hydrophobic cleft, formed by their hexameric ring structure, that binds the carrier proteins. The termini of the six subunits serve as “tentacles” which constrict the bound carriers in a clamp-like structure that prevents their misfolding (Webb et al. 2006; Weinhäupl et al. 2018).

Tim22 is the essential and central subunit of the TIM22 complex. Tim22 is structurally related to the central subunits of the TIM23 complex, Tim17 and Tim23 (Qi et al. 2021; Zhang et al. 2021). Though, the other subunits of the TIM22 complex (Tim54, Tim18 and Sdh3 in yeast; TIM29 and AGK (acyl glycerol kinase) in humans) have no equivalents in the TIM23 translocase (Koehler et al. 2000; Kang et al. 2016, 2017; Stiller et al. 2016; Valpadashi et al. 2021). A specialized small Tim complex is bound to the IMS-facing side of the TIM22 complex, consisting of Tim9, Tim 10 and Tim12 (Tim10b in humans) (Weinhäupl et al. 2018). This ring-shaped chaperone complex presumably feeds the carriers into the inner membrane via a lipid-Tim22 interface. The structure of Tim22 resembles that of Tim17 (Qi et al. 2021) and both translocases apparently work mechanistically in an equivalent way, using half-channel-like structures that are laterally open to the lipid bilayer, however, the full mechanism remains to be elucidated. In the membrane, the newly inserted carrier proteins fold into their three-dimensional native structure. The binding of specific membrane lipids, in particular cardiolipin molecules, supports carrier folding and stabilizes their conformation (Senoo et al. 2024).

## 4 Carriers can be proteotoxic

**Figure 3:**
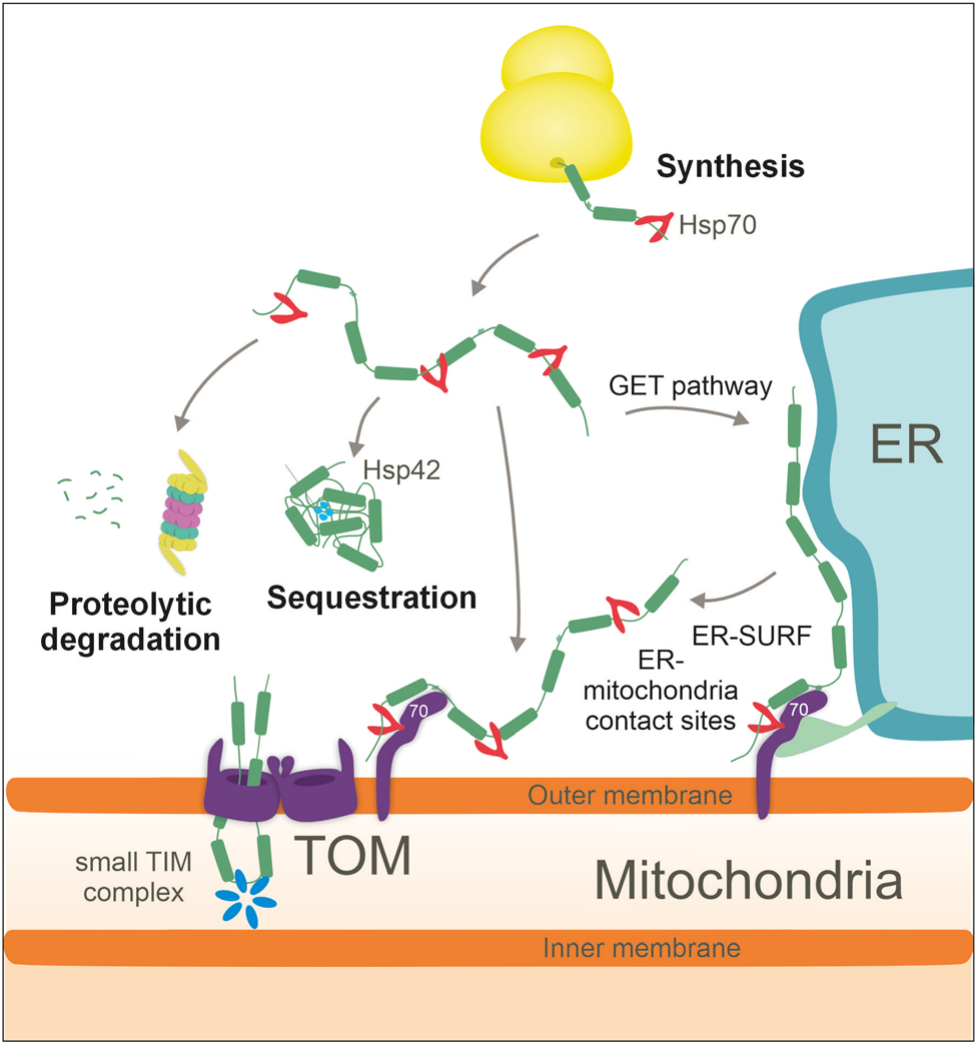
Carriers are under surveillance of the cytosolic quality control system. Cytosolic factors control the early steps of the biogenesis of carrier proteins. If the synthesis of carrier proteins exceeds the capacity of the import machinery, carrier proteins can be degraded by the proteasome or packaged into cytosolic aggregates in an Hsp42-dependent reaction. Alternatively, carriers can be targeted to the ER surface by the GET complex from where they transfer to the TOM complex via the ER-SURF pathway or to get degraded by ER-associated degradation.

Carrier proteins are hydrophobic and prone to form insoluble aggregates (Liu et al. 2019; Xiao et al. 2021). Carrier proteins lack N-terminal targeting signals and the time they spend in the cytosol before reaching mitochondria is unknown but it is probably very brief (Figure 3). Upon deletion of Tom70, the overexpression of carriers inhibits cell growth, suggesting that the rapid transfer from ribosomes to mitochondria reduces carrier toxicity (Backes et al. 2021). Moreover, mutations in carrier proteins which slow down their targeting to mitochondria can be detrimental for cells (Wang and Chen 2015). The reason for this toxicity which is called mPOS (mitochondrial precursor over-accumulation stress) is not known (Wang and Chen 2015). It was suggested that carriers can form stalled translocation intermediates (so-called cloggers) which inhibit mitochondrial protein import (Coyne et al. 2023). The ATP/ADP carriers are highly abundant proteins so that their efficient translocation is crucial to avoid “clogging”, i. e. competitive inhibition of the TOM complexes. In addition, non-imported carriers can accumulate in other cellular locations and compromise important functions. Indeed, non-imported carriers were found to accumulate on the ER membrane (Xiao et al. 2021). Components of the guided entry of tail-anchored proteins (GET) facilitate their targeting to the ER potentially to prevent their aggregation and to facilitate their transfer to the TOM complex via the ER-mitochondria contact sites on the ER-SURF targeting pathway (Koch et al. 2024; Hansen et al. 2018; Xiao et al. 2021). The overexpression of cytosolic chaperones which associate co-translationally with nascent chains, such as Ssb1, Ssb2 and Zou1, suppresses carrier toxicity, suggesting that these factors play a role in preventing carrier-induced proteotoxicity (Wang and Chen 2015).

## 5 The physiological role of carriers

Mitochondria play a central role in numerous metabolic processes, necessitating the transport of various substrates into and out of the organelle. The members of the mitochondrial carrier family facilitate these essential transport functions, making them crucial for a wide range of processes, including the tricarboxylic acid (TCA) cycle, oxidative phosphorylation, and the synthesis of DNA, RNA, and proteins within the mitochondria (Palmieri and Monne 2016; Palmieri et al. 2000; Ruprecht and Kunji 2020). Additionally, they are involved in metabolic pathways such as gluconeogenesis, lipogenesis, and urea synthesis. This chapter aims to provide an overview of the physiological roles of mitochondrial carriers, with a specific focus on *Saccharomyces cerevisiae*.

### 5.1 Nucleotide transport

Among the SLC25 family, the nucleotide carriers are responsible for the transport of nucleotides across the mitochondrial inner membrane. Nucleotides are essential for several processes, including maintaining genetic material, regulating cellular activities and metabolic processes, and maintaining cellular energy levels.

ATP serves as the universal energy “currency” of cells. In cells of animals and fungi, the cytosol typically contains about 2–5 mM of ATP, and much lower levels of ADP, AMP and adenosine (Takaine et al. 2022). In respiring cells, about 90 % of the ATP is synthesized in the mitochondrial matrix by the ATP synthase complex, whereas most ATP is hydrolysed outside mitochondria. Thus, special transporters are required to transport ATP out of mitochondria and maintain the cellular energy balance. For this transport of ATP, ADP and phosphate, nucleotide and phosphate carriers cooperate in the inner membrane of mitochondria.

The genome of baker’s yeast encodes three isoforms of ADP/ATP carriers, named **Aac1**, **Pet9** (or Aac2) and **Aac3** (Lawson and Douglas 1988). They are orthologues of the human adenine nucleotide transporter ANT, from which also several distinct isoforms exist (Stepien et al. 1992) (Table 1). With about 190,000 copies per cell under respiring conditions, Pet9 is one of the most abundant proteins of the inner mitochondrial membrane (Morgenstern et al. 2017). The other two isoforms, Aac1 and Aac3, are expressed at low levels or only under anaerobic conditions (Drgon et al. 1991). All three isoforms are functionally equivalent and can replace each other in deletion mutants. However, under physiological conditions, they may have specific preferences for the ATP-out, ADP-in transport, for example, under respiring conditions or in glucose-grown non-respiring cells (Ruprecht et al. 2019).

The ATP/ADP carriers closely cooperate with the phosphate carriers for each ATP/ADP exchange, in which one phosphate needs to be transported. In yeast, two isoforms of phosphate carriers exist, **Mir1** and **Pic2**. Both can catalyze the cotransport of a proton and phosphate (Hamel et al. 2004), however, under physiological conditions, Mir1 carries out phosphate transport while Pic2 is a copper transporter (Zhu et al. 2021). Mir1 has initially been proposed to function as a protein translocase in the inner membrane (Murakami et al. 1990) but this turned out not to be the case (Zara et al. 1996).

The ATP/ADP carriers do not lead to net import of nucleotides into mitochondria. In order to increase the levels of adenine nucleotides in the matrix, which is of course crucial for cell propagation, mitochondria use the ATP-magnesium/phosphate exchanger **Sal1** (Kucejova et al. 2008). Sal1 activity is regulated by calcium for which Sal1 exposes EF hand motifs into the IMS. Thus, on a physiological level, Pet9 appears to serve as the major adenine nucleotide exchanger and Sal1 as the main adenine nucleotide importer. However, the functions of both proteins are not absolutely distinct and in fact partially overlap, so that single mutants are viable and only a double deletion of Pet9 and Sal1 is lethal (Chen 2004).

The GTP/GDP carrier **Ggc1** transports GTP, GDP, dGTP, and dGDP across the inner membrane. Deletion of Ggc1 leads to the loss of the mitochondrial genome indicating that Ggc1 is crucial for the net translocation of guanosine nucleotides.

The transport of pyrimidine (desoxy)nucleotides is carried out by **Rim2** and accordingly, null mutants of Rim2 lose their mitochondrial DNA and show growth defects on non-fermentable carbon sources (Marobbio et al. 2006). Interestingly, Rim2 also serves as iron transporter in the inner membrane and both of its functions, the transport of pyrimidines and that of iron, can be genetically separated using different Rim2 mutants (Knight et al. 2019).

The peroxisomal carrier **Ant1** imports ATP in exchange for the AMP which is a product of beta-oxidation of fatty acids (Palmieri et al. 2001b; van Roermund et al. 2001). It is not required for mitochondrial function and targeted to peroxisomes by the Pex19-Pex3 pathway. In the peroxisome membrane it cooperates with a fraction of the phosphate carrier Mir1 which is presumably dually localized to mitochondria and peroxisomes, even though this has only been shown for *Hansenula polymorpha* cells (Pedersen et al. 2024).

### 5.2 Metabolite transport

The mitochondrial carrier proteins transport a range of solutes which are crucial for cellular metabolism. Carriers are crucial for catabolic reactions such as the export of newly synthesized amino acids from the matrix to the cytosol. The metabolite carriers can be subcategorized into groups according to their substrates, but these groups are not well defined, and some have a rather broad substrate specificity.

The TCA cycles relies on the supply of pyruvate that is transported from the cytosol into the matrix. The pyruvate transporters were only identified recently (Bricker et al. 2012; Herzig et al. 2012). Three mitochondrial pyruvate carrier subunits exist in yeast (Mpc1-3) as well as in humans (MPC1, MPC1L and MPC2). They do not belong to the carrier SLC25 family and only contain three transmembrane domains. They form heterodimers so that again six transmembrane domains in the complex constitute the functional unit, which however in primary structure is completely unrelated to that of SLC25 carriers (Sichrovsky et al. 2025). Moreover, they use the “carrier pathway” for biogenesis and are inserted into the inner membrane by the TIM22 complex (Gomkale et al. 2020; Rampelt et al. 2020).

Several metabolites of the TCA cycle are exchanged with pools in the cytosol via carrier-mediated transport reactions:

**Sfc1** transports succinate across the inner membrane using fumarate as the counter substrate (Palmieri et al. 1997b). It is essential for gluconeogenesis as it provides the cytosol with oxaloacetate upon growth on ethanol or acetate.

**Dic1** transports dicarboxylates using phosphate as co-substrate and predominantly supplies the mitochondrial matrix with basic levels of aspartate, glutamate, fumarate, citrate, oxoglutarate, oxaloacetate and other intermediates of the TCA cycle (Palmieri et al. 1999). It thereby prevents the depletion of the TCA cycle intermediates and allows the cells to rapidly switch from fermentation to respiration, when the external glucose levels drop.

**Ctp1** imports citrate in exchange for cytosolic malate which, upon oxidation to oxaloacetate, reduces NAD^+^ to NADH in the matrix thereby feeding the respiratory chain (De Blasi et al. 2024). Mutants lacking Ctp1 show reduced growth rates on non-fermentable carbon sources.

**Yhm2** transports dicarboxylates (citrate, oxoglutarate, oxaloacetate, succinate, and fumarate), as some of the carriers above but has yet another physiological role: it is part of the citrate-oxoglutarate NADPH shuttle, a pathway that uses electrons from matrix NADPH to replenish NADPH levels in the cytosol. In this sense, it is of major relevance to maintain a reduced cytosol upon oxidizing conditions (Castegna et al. 2010). Accordingly, Yhm2 is critical to keep cytosolic thiol-dependent peroxidases and glutathione pools reduced (Calabrese et al. 2017).

**Odc1 and Odc2** isoforms of the oxodicarboxylate carrier transport oxoglutarate and oxoadipate from the matrix to the cytosol for lysine and glutamate synthesis. The two isoforms are functionally equivalent, but their amounts differ depending on the growth conditions; Odc1 is at higher levels in respiring cells while Odc2 is at higher levels under anaerobic conditions (Palmieri et al. 2001a). Overexpression of Odc1 can suppress the induction of the retrograde signaling pathway which responds to mitochondrial dysfunction. Modulation of Odc1 activity was therefore proposed as a potential strategy for the treatment of patients suffering from metabolic complications caused by mitochondrial DNA mutations (Su et al. 2019).

**Oac1,** the oxaloacetate carrier transports oxaloacetate, sulfate, thiosulfate and isopropylmalate. Since Oac1 is important for the synthesis of leucine, Oac1-deficient mutants require the supplementation of leucine for efficient growth (Marobbio et al. 2008).

**Agc1 and Agc2** (aralar 1 and citrin in humans) are carrier proteins that facilitate the transport of amino acids across the inner membrane. These are required as building blocks for the protein synthesis in the mitochondrial matrix but also used for catabolic and anabolic reactions (Kunji et al. 2020). Specifically, Agc1 and Agc2 serve as transporters for aspartate and glutamate exporters. They are part of the malate-aspartate shuttle and critical for efficient gluconeogenesis, purine and pyrimidine metabolism, ornithine synthesis and the urea cycle. Agc1 is much larger than other carrier proteins as it has a 600 residue-extension on its N terminus which may have a regulatory role. Mutations in human citrin lead to citrullinemia or citrin deficiency, a highly prevalent mitochondrial disease (Tavoulari et al. 2024).

**Ymc1 and Ymc2** are also glutamate-transporting carriers which may play roles in fatty acid metabolism (Porcelli et al. 2018; Trotter et al. 2005).

**Hem25** is a glycine carrier required for mitochondrial heme biosynthesis (Lunetti et al. 2016) and is also required for the transport of isopentenyl pyrophosphate which serves as critical precursor molecule for coenzyme Q (Tai et al. 2023). Patients with mutations in the human homolog SLC25A38 suffer from reduced heme and pyridoxal 5′-phosphate levels causing congenital sideroblastic anemia (Pena et al. 2025).

**Ort1,** the ornithine carrier is critical for the synthesis of arginine (Palmieri et al. 1997a).

### 5.3 Transport of NAD^+^, FAD and other cofactors

Electron donors such as NADH and FADH_2_ are essential for mitochondrial respiration. The carriers **Ndt1 and Ndt2** transport NAD^+^ across the inner membrane (Todisco et al. 2006). They also transport (d)AMP and (d)GMP but not NADH, NADP^+^, or NADPH. Their human counterparts as NAD^+^ transporters are SLC25A51 and SLC25A52 (Girardi et al. 2020; Kory et al. 2020; Luongo et al. 2020; Ouyang et al. 2021).

**Flx1** serves as transporter for FAD (Tzagoloff et al. 1996). Flx1 is also crucial for the activity and stability of the succinate dehydrogenase complex, and the intramitochondrial flavin levels apparently regulate Sdh1 biogenesis (Bafunno et al. 2004; Moosavi et al. 2019).

Carrier proteins also mediate the transport of adenosine 5′-phosphosulfate (**Mrx21**), coenzyme A (**Leu5**), thiamine (**Tpc1**), and S-adenosylmethionine (**Sam5**) across the inner membrane.

### 5.4 Transport of metals and other ions

The function of many proteins relies on the binding of metals and other ions. The mitochondrial matrix can serve as sink or buffer for ions. The transport across the mitochondrial inner membrane therefore is of importance for the ion homeostasis of the cell. The transport of calcium from the cytosol to the matrix is facilitated by the calcium uniporter (MCU), which is not a member of the SLC25 carrier family (Kirichok et al. 2004; De Stefani et al. 2011, 2016). The MCU is ubiquitously present in animal cells but absent in fungi.

Magnesium is imported into mitochondria by the magnesium transporters Mrs2 and Lpe10, which are not members of the SLC25 carrier family. The ATP carrier **Sal1,** which is a member of the SLC25 carrier family, imports ATP exclusively if it is bound to magnesium. The export of magnesium is carried out by the carrier protein **Mme1** (Cui et al. 2015). Under conditions of transient magnesium depletion, Mme1 exports l magnesium from mitochondria to the cytosol and thereby protecting cells against magnesium starvation (Cui et al. 2015).

The transport of iron into mitochondria is an essential process as enzymes of the matrix are absolutely necessary for the synthesis of cellular iron-sulfur clusters (Lill and Freibert 2020). Moreover, iron is required for heme synthesis and for different iron-binding enzymes. The carriers **Mrs3** and **Mrs4** serve as iron transporters across the inner membrane (Froschauer et al. 2009; Muhlenhoff et al. 2003; Wiesenberger et al. 1991). They are essential under iron starvation, however, in iron-rich media other carriers (e.g. Rim2) still transport enough iron across the inner membrane even if Mrs3 and Mrs4 are absent. Mrs3 and Mrs4 also partially overlap with the function of the copper transporter **Pic2** (Vest et al. 2016), and these carriers might also facilitate the transport of other metals.

**Fsf1** is another inner membrane protein with presumed function in iron metabolism, but its substrate is still unknown; Fsf1 is no member of the carrier family but belongs to the group of sideroflexins (Cunningham and Rutter 2020; Kory et al. 2018; Miotto et al. 2007).

## 6 Outlook

Over the last 20 years, a function was assigned to most of the 35 carrier proteins of yeast cells. This proved to be rather difficult as the deletion of most carriers uniformly leads to respiration incompetence and does not reveal much about the specific biochemical function of carriers. While the reconstitution into liposomes was extremely helpful to define the substrate spectrum of individual carriers, it often did not provide much information about their specific physiological role. The example of the dicarboxylate carriers showed that transporters of similar substrate spectrum can have very different physiological roles in anabolism, catabolism, signaling or redox control. Another question that has not been fully addressed, is the interaction such carriers with other proteins within the membrane which could reveal novel modes of regulation.

Many recent studies identified defects in individual carrier proteins as a cause of human diseases (Palmieri et al. 2020). Since many human carriers have direct counterparts in yeast, which often even can be functionally exchanged, yeast serves as excellent model system to study the biology of carriers and to analyze the consequence of patient-derived mutations in carrier proteins. Many aspects of the biogenesis and physiology of carriers are still unclear and await to be discovered.
